# Relationship between cortical microinfarcts and cognitive impairment in Alzheimer's disease

**DOI:** 10.1590/S1980-57642012DN06030004

**Published:** 2012

**Authors:** Benito P. Damasceno

**Affiliations:** Professor of the Department of Neurology, Medical School, State University of Campinas (UNICAMP), Campinas SP, Brazil.

**Keywords:** Alzheimer's disease, vascular cognitive impairment, dementia, microinfarcts, cerebral amyloid angiopathy, neurofunctional networks

## Abstract

Cerebrovascular disease and AD pathology co-exist in most dementia cases, and microinfarcts (MIs), particularly if cortical and multiple, play an additive and independent role in AD cognitive impairment. The main cause of cortical MIs is chronic cerebral hypoperfusion but occlusive vascular diseases, embolism and blood-brain barrier disruptions, isolated or combined, may also play a role. The precise mechanisms by which MIs cause cognitive impairment are not well known, but one plausible explanation is that they are widespread and accompanied by diffuse hypoperfusion, hypoxia, oxidative stress and inflammation, particularly in the watershed areas of the tertiary association cortex, and hence could damage cognition networks and explain many of AD's cognitive and behavioral disturbances. Therefore, it is crucial to control vascular risk factors and avoid uncontrolled use of the antihypertensives, neuroleptics and other sedative drugs frequently prescribed to AD patients.

## Abstract (pt)

Doença cerebrovascular e patologia da doença de Alzheimer (DA) coexistem na maioria dos casos de demência, nos quais os microinfartos desempenham papel relevante, aditivo e independente. A principal causa de microinfartos corticais é a hipoperfusão cerebral crônica, porém doenças vasculares oclusivas, embolismo, lesões da barreira hematoencefálica, isolados ou combinados, podem também influir. O mecanismo preciso pelo qual microinfartos comprometem a cognição não são bem conhecidos, entretanto uma explicação plausível seria que eles são extensamente distribuídos e acompanhados de hipoperfusão difusa, hipóxia, estresse oxidativo e inflamação, principalmente nas zonas de fronteiras arteriais do córtex associativo terciário, e deste modo, eles poderiam lesar as redes neurais da cognição e explicar muitos dos transtornos cognitivos e comportamentais da DA. Por isso, é crucial prevenir os fatores de risco vascular e evitar o uso exagerado de anti-hipertensivos, neurolépticos e drogas sedativas frequentemente prescritas para pacientes com DA.

## INTRODUCTION

Cerebrovascular disease (CVD) is the second most common cause of cognitive impairment and dementia in the elderly, after Alzheimer's disease. CVD can cause a broad spectrum of cognitive, mental-behavioral and functional impairment ranging from very mild forms to severe dementia, thus constituting a "vascular cognitive impairment (VCI) - vascular dementia (VaD)" spectrum.

According to Di Legge & Hachinski,^1^ the concept of VCI refers to any cognitive impairment caused or associated with vascular risk factors, and also includes the link between CVD and Alzheimer's disease (AD). VCI and AD have a mixed etiology and share common risk factors for cognitive impairment, such as hypertension, diabetes mellitus, atherosclerotic disease, inflammation, and atrial fibrillation,^2-4^ hence the possibility to prevent both diseases. As highlighted by Di Legge & Hachinski,^1^ cognitive impairment related to CVD and AD share common elements:

[1] stroke may precede, trigger, co-exist, or exacerbate AD-type cognitive impairment; and
[2] AD-associated cerebral amyloid angiopathy (CAA) impairs blood vessel function and can cause brain ischemia and cognitive impairment independent of stroke (see also Greenberg et al.).^5^

As yet there is no universally accepted diagnostic criteria for VCI, especially because the studies available have used different neuropsychological test batteries, neuroimaging criteria, and definitions of cognitive impairment.^4,6-8^ In order to tackle these methodological heterogeneities and pave the way for a consensus diagnosis, the National Institute of Neurological Disorders and Stroke (NINDS) and the Canadian Stroke Network (CSN) have recommended harmonization criteria concerning VCI clinical features, neuropsychology, neuroimaging, neuropathology, experimental models, genetics, biomarkers, and clinical trials.^9^

The most common cause of VCI is ischemia or infarct in the territories of small caliber arteries and arterioles (cerebral arteriolosclerosis or small-vessel disease), with isolated lacunar infarcts and diffuse, ischemic white matter lesions (leukoaraiosis) in periventricular and deep subcortical white matter.^10,11^ Other common neuropathological causes are:

[1] large vessel disease with single, strategic (e.g., thalamic) or multiple, cortico-subcortical infarcts (multi-infarct dementia);
[2] severe hypoperfusion state, with maximal damage in hippocampal CA1 neurons, cortical watershed areas and deep white matter;
[3] hereditary vasculopathy (e.g., cerebral autosomal dominant arteriopathy with subcortical infarcts and leukoencephalopathy, CADASIL); and
[4] CAA with haemorrhage or microbleeds.^11,12^ Cortical microinfarcts also contribute to the cognitive decline in individuals at high risk for Alzheimer's dementia.^13^

In experimental models of VCI in rodents and primates, neuropathological changes comprise microinfarcts, diffuse white matter lesions, hippocampal neuronal loss, focal ischemic lesions and micro-haemorrhages, with subsequent deficits mostly in working and reference memory, as shown in a systematic review of 107 studies by Jiwa et al.^11^ These models consist of brief global ischemic insults or chronic global hypoperfusion; embolic lesions; chronic hypertension; strategic or multiple ischemic lesions, and generalised vasculopathies (e.g., in transgenic mice models of CAA and CADASIL).

Thus, as yet we have an incomplete understanding of the relationships between the pathophysiology and neuropsychological features of VCI and between vascular disease and Alzheimer's neurodegenerative brain changes. In this regard, the aim of this article was to review the literature focusing on the relationship between microinfarcts (MIs) and Alzheimer's dementia, given the additive and independent role they play in AD cognitive impairment, even in the 15% of cases without macroinfarcts, lacunes, atherosclerosis, or CAA.^14^ "Focal" ictal vascular syndromes caused by macroinfarcts or lacunes, such as aphasia, apraxia, and agnosia, will not be dealt with, since there are controversies regarding their inclusion or not in the concept of VCI. Publications retrieved from electronic databases (Medline, Pubmed), scientific articles, systematic reviews, and meta-analyses were surveyed. It is proposed that microinfarcts could damage cognition networks and explain most of AD neuropsychological features, due to the widespread distribution and association of MIs with diffuse hypoperfusion, hypoxia, oxidative stress and inflammation, particularly in the watershed areas of the tertiary association cortex.

## MICROINFARCTS, COGNITIVE IMPAIRMENT AND ALZHEIMER'S DEMENTIA

**Microinfarcts and their physiopathology.** In a systematic review of neuropathological studies involving 10,515 people, Brundel et al.^15^ found that MIs are common in patients with vascular dementia (weighted average 62%), Alzheimer's disease (43%), demented patients with both Alzheimer-type and cerebrovascular pathology (33%), and even in nondemented older individuals (24%). Cortical MIs and, to a lesser degree, periventricular demyelination (but not subcortical MIs) are associated with cognitive decline, particularly in individuals at high risk for dementia.^16,17^

Microinfarcts (MIs) are well-delimited microscopic regions of cellular death or tissue necrosis, sometimes with a central fluid-filled cavity, and a size (diameter of the maximum extent) varying from 0.1 to 2.9 mm.^17,18^ Most microinfarcts have a size (0.2-1.0 mm) below the lower limit of spatial resolution for magnetic resonance imaging (MRI) at conventional field strengths (1.5-3.0 Tesla), but can be detected by using ultrahigh-field (7 Tesla) MRI.^19^

MIs are found mainly in watershed areas, in the cortical borderzones between the territories of anterior and middle, and posterior and middle cerebral arteries (frontal and parieto-occipital regions, respectively), and are more numerous in the parietal-occipital region.^14,20^

In AD, the most important risk factor for the genesis of watershed cortical MIs is cerebral amyloid angiopathy (CAA), which is characterized by deposition of β-amyloid (especially the soluble subspecies βA40) in the media and adventitia of small arteries and capillaries of the leptomeninges and cerebral cortex, with subsequent capillary occlusion and decreased blood flow.^21,22^ CAA-associated microbleeds are also frequently found and could further contribute to impairment of cognitive function, even in elderly individuals without dementia.^23,24^ The main mechanism for cortical MIs is chronic cerebral hypoperfusion with hypoxia, oxidative stress, and inflammation, but occlusive vascular diseases (e.g., atherosclerosis, thromboangiitis obliterans), embolism, and blood-brain barrier disruptions, isolated or combined, may also play a role.^18,25^ The selective distribution of cortical MIs in watershed cortical areas, even in the 15% of AD cases without atherosclerosis and CAA, indicates that their main cause is cerebral hypoperfusion (usually due to arterial hypotension), which may be exacerbated by the use of antihypertensives, neuroleptics and other sedative drugs frequently prescribed to AD patients.^14,25^

**Relationship between microinfarcts and cognitive impairment.** As shown by several studies, the burden and location of macro- and microinfarcts are significantly associated with cognitive decline.^16,17,26^ In a prospective community-based autopsy study, Arvanitakis et al.^17^ used neuropsychological tests to examine the influence of quantity and location of MIs on five cognitive systems. They found that MIs were associated with lower levels of semantic memory and perceptual speed, with a lesser association with episodic memory, and no relationship with working memory or visuospatial abilities. Only in the analyses of location were cortical MIs associated with visuospatial abilities. Of note, subcortical MIs were not associated with any of the cognitive systems.

Autopsy studies show that MIs have an additive or synergistic effect in combination with AD pathology and, in spite of being small and "silent", they contribute to cognitive impairment and dementia, particularly if multiple and cortical.^17^ Although MIs are highly frequent in brains of people without dementia, their presence in the cerebral cortex is an independent predictor of worse cognitive function and dementia even in patients with low levels of neurofibrillary tangles (Braak stage < III) and without macroinfarcts or lacunes.^18,27-29^ Interestingly, in multiple sclerosis, another degenerative-inflammatory disease as yet considered to be mostly subcortical, highly frequent small cortical lesions constitute the main cause of cognitive decline and social-occupational dysfunction.^30,31^

The precise mechanisms by which MIs cause cognitive impairment are not well known. As one plausible explanation, we propose that, given their widespread distribution and association with diffuse hypoperfusion, hypoxia, oxidative stress and inflammation, particularly in the watershed areas of the tertiary association cortex, they could damage cognition networks.

Cognitive functions such as attention, language, visuospatial abilities and memory are complex systems or networks comprising various basic mental operations or processes organized in a dynamic assembly of interconnected brain regions, each region making its specific contribution to the functioning of the system as a whole.^32-35^ According to this concept, every mental act (e.g., naming the picture of an object, solving a problem) is carried out by a dynamic neurofunctional network whose psychological and cerebral components change from moment to moment insofar as each task or operation switches one for another, with each mental act requiring a different ensemble of cognitive processes suitable for achieving the objective of the task. Within such a network, there are a few crucial nodes, so-called convergence-divergence zones^36^ or connector hubs,^37^ which:

[1] integrate the functions of distant microcircuits by means of simultaneous synthesis;
[2] contain the neural substratum (basic operations) of different complex mental acts; and
[3] can thus belong to various partly overlapped neuronal networks.^35^

This parallel distributed processing of cognitive functions make them more vulnerable to dysfunction by widespread and diffuse lesions. Dysfunction of any connector hub due to stroke or neurodegenerative pathological process, as in AD, may cause "focal" neuropsychological syndromes or even dementia, as happens in cases with strategic infarct in the thalamus (thalamic dementia) or in the left inferior parietal region (angular gyrus syndrome simulating Alzheimer's dementia). Indeed, AD and other neurodegenerative diseases (behavioral variant frontotemporal dementia, semantic dementia, progressive nonfluent aphasia, and corticobasal syndrome) have been thought to target specific neuronal networks,^38^ and a breakdown of functional connectivity in the default mode network typically occurs even in the early phase of AD.^39,40^

As a corollary of the network concept, different components of the same complex mental act or function can be impaired by lesions in different regions or in their interconnecting pathways. For example, one of the most common linguistic symptoms in AD and in left hemisphere stroke - anomia or word finding difficulties, may result from damage to any of a variety of brain regions (temporal, parietal, frontal, or thalamic). An apparently simple mental act such as naming the picture of a horse would require:

[1] processes for abstracting the features that allow recognition of the object (its visual perception) from the visual stimulus;
[2] access to the meaning of horse (its semantic representation);
[3] access to the learned pronunciation of horse (its phonologic representation); and
[4] the phono-articulatory motor programming required to articulate the word "horse",^41^ with each of these processes occurring in different interconnected brain regions.

Even access to the meaning itself (semantic representation or semantic memory) entails a complex co-activation of all of the relevant features of the object (visual, smell, motion, how humans use it, etc.), including category membership (animate being, animal), all of these features tied together via a synchronized 30 Hz gamma rhythm neuronal firing in interconnected cortical regions, mediated by the thalamus,^42^ and this could be the reason why thalamic infarcts can independently contribute to cognitive impairment and dementia.^43^

Because of their systemic organization, cognition and behavior are highly vulnerable to multifocal lesions. Thus, we speculate that the widespread and diffuse damage caused by MIs in the watershed areas (WAs) of association cortex could, at least in part, produce many of AD's cognitive and behavioral disturbances, although it cannot explain the impairment of episodic memory associated to entorhinal-hippocampal dysfunction, typical of the disease. For this dysfunction, a more plausible explanation proposed by Kapogiannis & Mattson^44^ is that hippocampal-entorhinal neurons, particularly the hippocampal pyramidal cells, have the highest energy requirements of any neurons in the brain and are therefore more vulnerable when metabolic needs are not met, for example, during high levels of excitation under conditions of age-related and disease-related mitochondrial impairment and oxidative stress. As suggested by these authors, AD-related early accumulation of βA oligomers in synapses might result in tau hyperphosphorylation and aggregation in neurons of entorhinal cortex (layers II-III, and IV) and subiculum, with neuronal death in these structures and secondary hypometabolism and atrophy in other interconnected transmodal cortices, mostly in the posterior medial cortex (posterior cingulate cortex and precuneus), which is a crucial connector hub for episodic retrieval and semantic processing in the default functional (resting) state of the brain. Hence, synaptic dysfunction mediates transneuronal spread of AD, which targets its specific neural networks.^38,44^

### Cognitive-behavioral syndromes related to MI distribution in cortical WAs

*Anterior WAs* - MIs in anterior WAs (borderzones of middle and anterior cerebral artery territories), particularly in the middle-superior frontal gyrus region near the prefrontal-premotor border, could cause a disexecutive syndrome, especially for planning and carrying out complex tasks, impairment of working memory, and problem-solving difficulties; and in predominantly left-sided lesions, signs of transcortical motor aphasia, with poor and nonfluent, frequently echolalic, verbal output. In AD, the aphasic syndrome is almost always fluent, and more often an anomic (semantic) or transcortical sensory aphasia, probably due to the predominance of MIs in the posterior WA areas. Mesulam^45^ warns that nonfluent aphasias are extremely rare in AD and should raise the possibility of an alternative diagnosis (see also Price et al.).^46^ Inferior lesions, in the prefrontal medial-orbital border, could contribute to loss of initiative (apathy), generalized disinhibition with loss of insight, self-criticism and self-control, failures in tests of theory of mind and empathy, disorder of emotion regulation, errors of social-moral judgement, and personality changes.

*Posterior WAs* - In posterior WAs (borderzones of middle-posterior and middle-anterior cerebral arteries), the inferior (occipital-temporal) lesions, especially to the left side, could cause a visual-verbal disconnection syndrome, with apperceptive visual agnosia or optic aphasia (visual anomia), characterized by the inability to recognize or name seen objects or their pictures, yet with normal recognition or naming of the same objects when they are touched or verbally described.

Superior-middle (occipital-parietal) lesions, near the posterior-inferior parietal region, could explain many of AD's visuospatial disorders, such as simultanagnosia, right-left and topographical disorientation, eye-hand discoordination, with difficulty in reaching a target under visual guidance (optic ataxia), and even a full Balint's syndrome; and in predominantly left-sided lesions, an ideomotor, ideational, and visuoconstructive apraxia, as well as acalculia and aphasia. The aphasia in AD is characteristically fluent, of the anomic type (Luria's semantic aphasia) in mild cases, or transcortical sensory (more rarely Wernicke-like) aphasia in more advanced cases, with poor comprehension, paraphasic spontaneous speech, increased use of empty words, and relatively preserved syntax and phonology, showing similar difficulties in reading and writing.^47-49^ All these aphasic features can be explained by dysfunction in or near the posterior WAs of the language dominant hemisphere, and indicate relative intactness of the more central, perisylvian territory of the middle cerebral artery.

*Inferior WAs* - MIs in the inferior WA (borderzones of middle and posterior cerebral arteries), mainly in the left inferolateral temporal lobes, may impair semantic memory, especially in naming and categorization tasks. Alzheimer's disease is the most common clinical disorder disrupting semantic memory, with more severe cases being unable to name an item when it is described and also unable to describe an item when they are given its name.^50^ The naming difficulty is partially segregated in the lateral temporal lobe depending on the conceptual category (e.g., person, animal, tool) to which the entity belongs,^51^ and it is more difficult to name basic level (e.g., house, dog) and unique entities (e.g., White House, rocking chair), which require finer-grained discrimination and access to more information than needed to name higher level entities (e.g., animal, fruit).^52^

**Conclusion.** CVD and AD-pathology co-exist in most dementia cases. In this regard, not only macroinfarcts, but also MIs play an additive, synergistic and independent role in AD cognitive impairment, even in the 15% of cases without atherosclerosis or cerebral amyloid angiopathy. The precise mechanisms by which MIs cause cognitive impairment are not well known, but one plausible explanation is that they are widespread and accompanied by diffuse hypoperfusion, hypoxia, oxidative stress and inflammation, particularly in the watershed areas of the tertiary association cortex, and hence could damage cognition networks thereby explaining many of AD's cognitive and behavioral disturbances. Since CVD and AD are often associated, it is crucial to control vascular risk factors and avoid uncontrolled use of the antihypertensives, neuroleptics and other sedative drugs frequently prescribed to AD patients.

## References

[1] Di Legge S, Hachinski V (2010). Vascular cognitive impairment (VCI): progress towards knowledge
and treatment. Dement Neuropsychol.

[2] Cechetto DF, Hachinski V, Whitehead SN (2008). Vascular risk factors and Alzheimer's disease. Exp Rev Neurotherapeut.

[3] Viswanathan A, Rocca WA, Tzourio C (2009). Vascular risk factors and dementia: How to move
forward. Neurology.

[4] Merino JG, Hachinski V (2009). Vascular cognitive impairment: Evolution of the
concept. JINS.

[5] Greenberg SM, Gurol ME, Rosand J, Smith EE (2004). Amyloid angiopathyrelated vascular cognitive
impairment. Stroke.

[6] Zhou A, Jia J (2009). Different cognitive profiles between mild cognitive impairment
due to cerebral small vessel disease and mild cognitive impairment of
Alzheimer's disease origin. JINS.

[7] Delano-Wood L, Bondi MW, Sacco J (2009). Heterogeneity in mild cognitive impairment: Differences in
neuropsychological profile and associated white matter lesion
pathology. JINS.

[8] Sachdev PS, Chen X, Brodaty H, Thompson C, Altendorf A, Wen W (2009). The determinants and longitudinal course of post-stroke mild
cognitive impairment. JINS.

[9] Hachinski V, Iadecola C, Petersen RC (2006). National Institute of Neurological Disorders and Stroke -
Canadian Stroke Network Vascular Cognitive Impairment Harmonization
Standards. Stroke.

[10] Kalaria RN, Erkinjuntti T (2006). Small vessel disease and subcortical vascular
dementia. J Clin Neurol.

[11] Jiwa NS, Garrard P, Hainsworth AH (2010). Experimental models of vascular dementia and vascular cognitive
impairment: a systematic review. J Neurochem.

[12] Engelhardt E, Tocquer C, André C (2011). Vascular dementia: diagnostic criteria and supplementary exams.
Recommendations of the Scientific Department of Cognitive Neurology and
Aging of the Brazilian Academy of Neurology. Part I. Dement Neuropsychol.

[13] Kövari E, Gold G, Herrmann FR (2007). Cortical microinfarcts and demyelination affect cognition in
cases at high risk for dementia. Neurology.

[14] Suter OC, Sunthorn T, Kraftsik R (2002). Cerebral hypoperfusion generates cortical watershed microinfarcts
in Alzheimer disease. Stroke.

[15] Brundel M, de Bresser J, van Dillen JJ, Kappelle LJ, Biessels GJ (2012). Cerebral microinfarcts: a systematic review of neuropathological
studies. J Cereb Blood Flow Metab.

[16] Kövari E, Gold G, Herrmann FR (2007). Cortical microinfarcts and demyelination affect cognition in
cases at high risk for dementia. Neurology.

[17] Arvanitakis Z, Leurgans SE, Barnes LL, Bennet DA, Schneider JA (2011). Microinfarct pathology, dementia, and cognitive
systems. Stroke.

[18] Smith EE, Schneider JA, Wardlaw JM, Greenberg SM (2012). Cerebral microinfarcts: the invisible lesions. Lancet Neurol.

[19] Jouvent E, Poupon C, Gray F (2011). Intracortical infarcts in small vessel disease: a combined 7-T
postmortem MRI and neuropathological case study in cerebral
autosomal-dominant arteriopathy with subcortical infarcts and
leukoencephalopathy. Stroke.

[20] Okamoto Y, Ihara M, Fujita Y, Ito H, Takahashi R, Tomimoto H (2009). Cortical microinfarcts in Alzheimer's disease and subcortical
vascular dementia. Neuroreport.

[21] Smith EE, Greenberg SM (2009). β-amyloid, blood vessels, and brain
function. Stroke.

[22] Okamoto Y, Yamamoto T, Kalaria RN (2012). Cerebral hypoperfusion accelerates cerebral amyloid angiopathy
and promotes cortical microinfarcts. Acta Neuropathol.

[23] Van Norden AG, van den Berg HA, de Laat KF, Gons RA, van Dijk EJ, de Leeuw FE (2011). Frontal and temporal microbleeds are related to cognitive
function: the Radboud University Nijmegen Diffusion Tensor and Magnetic
Resonance Cohort (RUN DMC) Study. Stroke.

[24] Poels MM, Ikram MA, van der Lugt A (2012). Cerebral microbleeds are associated with worse cognitive
function: the Rotterdam Scan Study. Neurology.

[25] Miklossy J (2003). Cerebral hypoperfusion induces cortical watershed microinfarcts
which may further aggravate cognitive decline in Alzheimer's
disease. Neurol Res.

[26] Troncoso JC, Zonderman AB, Resnick SM, Crain B, Pletnikova O, O'Brien RJ (2008). Effect of infarcts on dementia in the Baltimore Longitudinal
Study of Aging. Ann Neurol.

[27] Snowdon DA, Greiner LH, Mortimer JA, Riley KP, Greiner PA, Markesbery WR (1997). Brain infarction and the clinical expression of Alzheimer's
disease. The Nun Study. JAMA.

[28] Kövari E, Gold G, Herrmann FR (2004). Cortical microinfarcts and demyelination significantly affect
cognition in brain aging. Stroke.

[29] Launer LJ, Hughes TM, White LR (2011). Microinfarcts, brain atrophy, and cognitive function: the
Honolulu Asia Aging Study Autopsy Study. Ann Neurol.

[30] Geurts JJ, Barkhof F (2008). Grey matter pathology in multiple sclerosis. Lancet Neurol.

[31] Amato MP, Ponziani G, Siracusa G, Sorbi S (2001). Cognitive dysfunction in early-onset multiple sclerosis: a
reappraisal after 10 years. Arch Neurol.

[32] Luria AR (1973). The working brain.

[33] Mesulam M-M (1998). From sensation to cognition. Brain.

[34] Mesulam M-M (2008). Representation, inference, and transcendent encoding in
neurocognitive networks of the human brain. Ann Neurol.

[35] Damasceno BP, Tsai J. P. (2008). Research on cognition disorder: methodological
issues. Leading-Edge Cognitive Disorders Research.

[36] Damasio AR (1989). Time-locked multiregional retroactivation: a systems-level
proposal for the neural substrates of recall and recognition. Cognition.

[37] Buckner RL, Sepulcre J, Talukdar T (2009). Cortical hubs revealed by intrinsic functional connectivity:
mapping, assessment of stability, and relation to Alzheimer's
disease. J Neurosci.

[38] Seeley WW, Crawford RK, Zhou J (2009). Neurodegenerative diseases target large-scale human brain
networks. Neuron.

[39] Greicius MD, Srivastava G, Reiss AL, Menon V (2004). Default-mode network activity distinguishes Alzheimer's disease
from healthy aging: Evidence from functional MRI. PNAS.

[40] Zhang HY, Wang SJ, Liu B (2010). Resting brain connectivity: changes during the progress of
Alzheimer's disease. Radiology.

[41] Hillis AE (2007). Aphasia: progress in the last quarter of a
century. Neurology.

[42] Hart J, Kraut MA (2007). Neural basis of semantic memory.

[43] White L, Petrovitch H, Hardman J (2002). Cerebrovascular pathology and dementia in autopsied Honolulu-Asia
Aging Study participants. Ann N Y Acad Sci.

[44] Kapogiannis D, Mattson MP (2011). Disrupted energy metabolism and neuronal circuit dysfunction in
cognitive impairment and Alzheimer's disease. Lancet Neurol.

[45] Mesulam M-M (2000). Principles of Behavioral and Cognitive Neurology.

[46] Price BH, Gurvit H, Weintraub S, Geula C, Leimkuhler E, Mesulam M (1993). Neuropsychological patterns and language déficits in 20
consecutive cases of autopsy-confirmed Alzheimer's disease. Arch Neurol.

[47] Hier DB, Hagenlocker K, Shindler AG (1985). Language disintegration in dementia: effects of etiology and
severity. Brain Lang.

[48] Cummings JL, Benson F, Hill MA, Read S (1985). Aphasia in dementia of the Alzheimer type. Neurology.

[49] Benke T, Andree B, Hittmair M, Gerstenbrand F (1990). Speech changes in dementia. Fortschr Neurol Psychiatr.

[50] Budson AE, Price BH (2005). Memory dysfunction. N Engl J Med.

[51] Damasio H, Tranel D, Grabowski T, Adolphs R, Damasio A (2004). Neural systems behind word and concept retrieval. Cognition.

[52] Balthazar MLF, Cendes F, Damasceno BP (2008). Semantic error patterns on the Boston Naming Test in normal
aging, amnestic mild cognitive impairment, and mild Alzheimer's disease: is
there semantic disruption?. Neuropsychology.

